# Impact of Computed Tomography Scans on the Risk of Thyroid Disease in Minor Head Injury Patients: A Population-Based Retrospective Cohort Study

**DOI:** 10.3390/ijerph17113873

**Published:** 2020-05-29

**Authors:** Shao-Lun Tsao, Yin-Yang Chen, Liang-Tsai Yeh, Jing-Yang Huang, Wen-Tyng Li, Shun-Fa Yang, Chao-Bin Yeh

**Affiliations:** 1 Department of Biomedical Engineering, Chung Yuan Christian University, Taoyuan 320, Taiwan; 117223@cch.org.tw (S.-L.T.); wtli@cycu.edu.tw (W.-T.L.); 2 Department of Anesthesiology, Changhua Christian Hospital, Changhua 500, Taiwan; 68990@cch.org.tw; 3 Institute of Medicine, Chung Shan Medical University, Taichung 402, Taiwan; jeff80329@hotmail.com (Y.-Y.C.); wchinyang@gmail.com (J.-Y.H.); 4 Department of Surgery, Chung Shan Medical University Hospital, Taichung 402, Taiwan; 5 Department of Medical Research, Chung Shan Medical University Hospital, Taichung 402, Taiwan; 6 Department of Emergency Medicine, School of Medicine, Chung Shan Medical University, Taichung 402, Taiwan; 7 Department of Emergency Medicine, Chung Shan Medical University Hospital, Taichung 402, Taiwan

**Keywords:** computer tomography, minor head injury, thyroid diseases

## Abstract

We investigated the association between head computed tomography (CT) scans and the risk of noncancer thyroid diseases in patients with minor head injury in a Taiwanese healthcare setting. For this retrospective population-based cohort study, the 2009–2013 Longitudinal Health Insurance Database was used to include patients with a minor head injury at admission or emergency visit between 2009 and 2013. Multivariate analysis with a multiple Cox regression model was applied to analyze the data. According to whether a CT scan was conducted within 14 days of admission, patients were divided into a CT scan group (n = 14,041) or a non-CT scan group (n = 34,684). No increased incidence of thyroid diseases was observed in the CT scan group regardless of the number of CT scans performed. The incidence rate ratio for one scan was 1.10 (95% confidence interval: 0.94–1.29) and for two or more scans was 1.09 (95% confidence interval: 0.93–1.28). In conclusion, this population-based cohort study showed that a head CT scan is not associated with increased risk of thyroid disease in patients with minor head injury. The short-term adverse effects on the thyroid could be mild when a regular CT scan is appropriately performed.

## 1. Introduction

Traumatic brain injuries (TBIs) frequently occur in Taiwan, causing serious short- and long-term sequelae in affected persons and socioeconomic burdens on their families and society. Approximately 2.8 million patients have experienced traumatic head injuries in the United States and TBIs caused more than 56,000 deaths every year from 2007 to 2013 [1]. In Taiwan, the incidence and death rate of TBI was 220.6/100,000 and 11.8/100,000 population in these years, respectively [2]. Minor head injury is considered when a patient with a TBI has a history of loss of consciousness, amnesia, or disorientation and a Glasgow Coma Scale score of 13–15 [3].

A non-contrast head computed tomography (CT) scan is useful for patients with TBI in the acute setting to identify brain injuries that require early neurologic evaluation and immediate medical or neurosurgical management. For patients with head trauma, the possibility of concurrent neck injury cannot be ruled out. A radiologist physician is required to adjust the scope of the CT scan to cover the C-spine where the thyroid is located. Therefore, the thyroid gland is inevitably exposed to radiation. Three criteria were developed and prospectively validated for patients with mild TBI and for those who were selected for a CT scan: the Canadian CT Head Rule (CCHR) [3], the New Orleans Criteria (NOC) [4], and the National Emergency X-Radiography Utilization Study II (NEXUS II) criteria [5].

A concern related to CT scanning is radiation exposure. The thyroid gland is vulnerable to radiation, which can induce thyroid cancer and other benign thyroid diseases, including thyroid nodules, autoimmune diseases, and other noncancer thyroid diseases [6,7]. However, whether radiation exposure during a head CT scan is correlated with thyroiditis remains controversial. Nagayama et al. reported that the effects of moderate- to low-dose radiation on thyroid autoimmunity and function are still inconsistent [8]. No data are available on the correlation between a head CT scan and thyroid diseases. Since a head CT scan is necessary for patients with moderate-to-severe head injury, we must pay more attention to potential thyroid diseases induced by radiation in patients with minor head injuries undergoing head CT scan. Therefore, we evaluated the impact of a head CT scan for patients with minor head injuries on the risk of thyroid diseases. Since thyroid dysfunction may be affected by several factors in patients with moderate-to-severe head injury, including brain injury [9,10,11], shock status [12], medication such as glucocorticoids and dopamine agonists [13], and massive transfusion [14], we focused on potential thyroid diseases induced by radiation in patients with minor head injuries undergoing a head CT scan. Therefore, the aim of this study was not to modify current guidelines, for which patients with minor head injuries should accepted a head CT scan, but to explore the risk of thyroid disease in these patients for early detection, surveillance and multidisciplinary management.

## 2. Material and Methods

### 2.1. Study Design and Data Source

In this retrospective cohort study, patients who were hospitalized or had an emergency visit for a minor TBI between 2009 and 2013 were selected for risk evaluation on thyroid disease. We used the Longitudinal Health Insurance Database 2010 (LHID 2010), a subdivision of Taiwan’s National Health Insurance Research Database (NHIRD). LHID 2010 comprises 1 million randomly sampled beneficiaries who were coved in Taiwan’s National Health Insurance (NHI) scheme in 2010. Under the NHI, all medical claims were regularly reviewed and reimbursed. The NHIRD contains information of beneficiaries’ demographics, all types of medical visits, codes for medical use, prescriptions, and diagnostic codes based on the International Classification of Diseases, 9th Revision, Clinical Modification (ICD-9-CM) system. Encrypted identification numbers of each patient were used to protect their privacy in this dataset. The Institutional Review Board of Chung Shan Medical University in Taiwan approved this study and informed consent was waived because the datasets were used anonymously and were deidentified before analysis.

### 2.2. Identification of Study Patients

**Figure 1:**
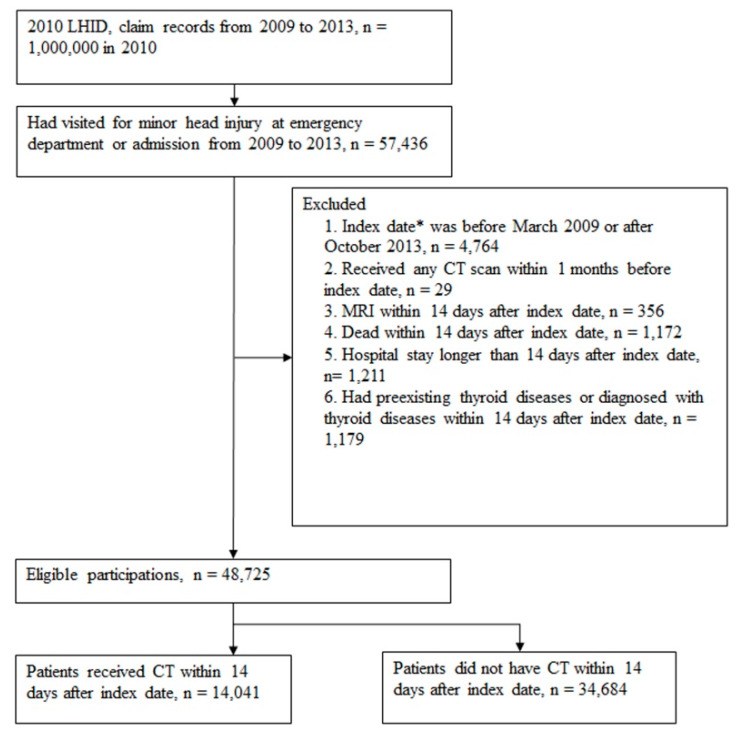
Flow chart for patient selection. * Index date is the date a patient visited for a minor head injury in the emergency department or at admission.

A total of 57,436 patients with TBI were identified using ICD-9-CM 850.x, 854.x, 910.x, 920.x, and 925.x in the admission and emergency data files. The index date was defined as the admission date and the patients were divided into CT scan and non–CT scan groups according to whether they underwent a CT scan within 14 days after the index date. We excluded individuals who (1) had an index date before March 2009 or after October 2013 (left truncation and right censoring data; n = 4764); (2) received any CT scan within 1 month before the index date (n = 29); (3) were subjected to an MRI within 14 days after the index date (n = 356); (4) died within 14 days after the index date (n = 1172); (5) had a hospital stay longer than 14 days after the index date (n = 1211); or (6) had preexisting thyroid diseases or were diagnosed as having thyroid diseases within 14 days after the index date (n = 1179). Hence, patients with a longer hospital stay, MRI, or mortality within 14 days were excluded to avoid the selection bias. After exclusion in the flow chart for patient selection (Figure 1), there were 14,041 and 34,684 patients with TBI that were included in the CT scan and non-CT-scan groups, respectively. The CT scan exposure cohort was subclassified into 2 groups according to the number of CT scans performed within 14 days: 1 CT scan and ≥2 CT scans.

### 2.3. Study Events and Follow-Up

We used a 14-day latency time to avoid possible observational bias; therefore, all individuals were at risk at 14 days after the index date. All ambulatory medical care and inpatient records for each patient were retrieved from their index admission until the end of 2013. The date of the first diagnosis of thyroid disease (ICD-9-CM 240.x–246.x) during the follow-up period was defined as the primary endpoint. Both cohorts were followed up until the occurrence of the study event, withdrawal from the NHI system or the end of 2013, whichever occurred first. The mortality rate within 6 months was also assessed to determine competing events.

### 2.4. Study Covariates

We considered event-related covariates, including demographics, such as sex, age, income, and urbanization; and comorbidity, such as diabetes mellitus: ICD-9-CM 250.x, hypertension: ICD-9-CM 401.x–405.x, chronic obstructive pulmonary disease: ICD-9-CM 490.x–496.x, coronary artery disease: ICD-9-CM 410.x–414.x, and cancer, ICD-9-CM ICD-9 140.x–208.x within 1 year before the index date. Hospital stays up to 1 month after the index date were also identified as a surrogate of the severity of the TBI.

### 2.5. Statistical Analysis

A chi-squared test was conducted to evaluate the heterogeneity of baseline characteristics among study groups. A time-to-event analysis was performed to calculate the incidence rate (per 1000 person-months) in study groups and the incidence rate ratio (IRR) was calculated to indicate the risk of thyroid disease in the CT scan group. Kaplan–Meier curves of the cumulative proportions for thyroid disease were plotted to observe the absolute risk over time. The log-rank test was used to compare the difference in disease-free probability among study groups. Multiple Cox regression analyses were used to estimate the adjusted hazard ratio (aHR) and 95% confidence interval (CI) of thyroid disease in the CT scan group after adjustment for age (on the index date), sex, comorbidities (within 1 year before the index date), and a hospital stay (within 1 month after the index date). SAS 9.4 (SAS Institute Inc., Cary, NC, USA) was used and the level of type 2 errors was set to 0.05.

## 3. Results

### 3.1. Characteristics of Study Subjects

We identified 48,725 patients who had visited the emergency department or had been admitted to the hospital due to a minor head injury from 2009 to 2013; 14,041 (28.82%) of them underwent a CT scan (Figure 1). Patients in the CT scan group were significantly older than those in the non-CT scan group. All the comorbidities were significantly different between the 2 groups. Moreover, 824 (5.87%) and 929 (6.62%) patients in the CT scan group received a second CT scan within 14 days and 2 months, respectively. The CT scan group had a longer hospital stay within 1 month and higher mortality within 6 months (Table 1) than the non-CT scan group.

### 3.2. Risk of Thyroid Diseases on Different Comorbidities and Other Conditions

**Figure 2:**
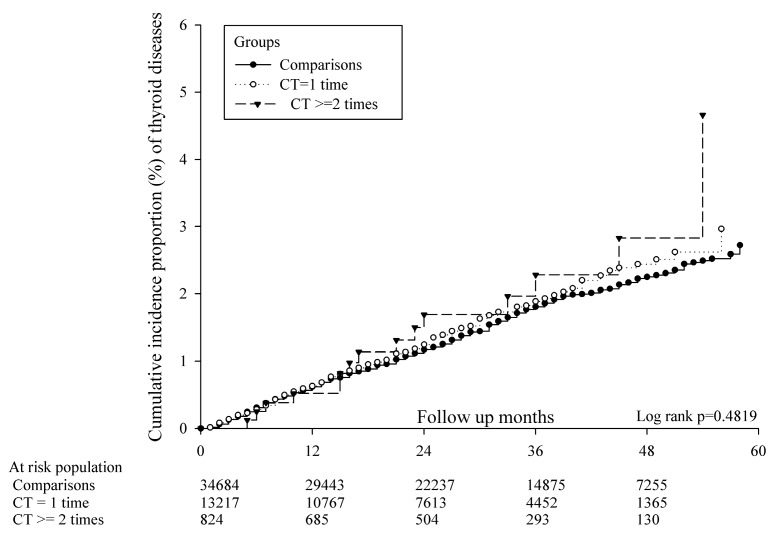
Kaplan–Meier curves of the cumulative proportion of thyroid disease.

Incidence of thyroid diseases was not higher in the CT scan group irrespective of the number of CT scans: the IRR for one scan was 1.10 (95% CI: 0.94–1.29) and for two or more scans was 1.09 (95% CI: 0.93–1.28) (Table 2). A Kaplan–Meier survival analysis revealed no significant difference in the cumulative incidence for the proportion of thyroid diseases in the CT scan group (log-rank test, *p* = 0.482; Figure 2). Compared with the non-CT-scan group, patients with either one or two or more CT scans were not associated with a higher risk of thyroid diseases (aHR: 0.933; 95% CI: 0.787–1.107 and aHR: 1.053; 95% CI: 0.618–1.794, respectively). The significant risk factors of thyroid disease were being female, COPD, and cancer (ICD-9 code 140–208) (Table 3).

We also performed a subgroup analysis for different types of thyroid diseases based on the ICD-9 code of diagnosis (Table 4). The risk of any thyroid disease was not increased in any of the three subgroups among patients in the CT scan group: for simple and unspecified nontoxic nodular goiter, the aHRs were 0.928 (95% CI: 0.698–1.235) in the one scan subgroup and 0.769 (95% CI: 0.241–2.457) in the two or more scans subgroup, respectively; for thyrotoxicosis with or without goiter, 0.850 (95% CI: 0.603–1.200) and 0.755 (95% CI: 0.232–2.457), respectively; and for acquired hypothyroidism and thyroiditis, 1.140 (95% CI: 0.758–1.608) and 1.796 (95% CI 0.699–4.619), respectively.

## 4. Discussion

Radiation is a well-known risk factor of thyroid disease or thyroid cancer. The present study is the first nationwide longitudinal population-based cohort study to evaluate the impact of head CT scans and the risk of thyroid disease in minor head injury patients. We found that CT scans (whether 1 or more scans) performed within 14 days of the injury does not increase the incidence of thyroid disease compared with patients not receiving a CT scan. The results of the Cox proportional hazards analysis of our study showed that COPD and cancer were significant risk factors; however, our study showed a neutral finding of CT scan and the comorbidities distribution did not affect our study results. A subgroup analysis for different types of thyroid diseases showed no increased risks of any thyroid disease in any of the three subgroups among patients with a minor head injury undergoing CT scans.

Typically, patients receive a radiation dose of 30–70 mGy during a head CT scan [15] and the dose is divided into the diagnostic part and superficial scatter [16]. Although the thyroid gland is a radiosensitive organ, no reliable data were available concerning the correlation between head CT scans and thyroid disease. Nagayama et al. reported inconsistent effects of moderate- to low-dose radiation on thyroid autoimmunity and function [8]. Ron et al. showed that significant effects of radiation were most often observed only under high radiation doses [6], but data concerning the risk of thyroid disease following radiation at lower doses are limited. In our study, head-CT-scan-induced radiation showed no correlation with prevalence of any thyroid diseases on the studied patient with a minor head injury. A previous study for evaluating the changes in the pattern of radiation-induced thyroid gland after radiotherapy showed a thyroid volume shrinkage and free thyroxine (fT4) level reduction decreasing from 0 to 30 months and reaching a relatively steady state after 36 months [17]. Therefore, the time period is comparable to the time setting for follow-up in this study.

Studies have focused more on the association between radiation and thyroid cancer. Mazonakis et al. assessed cancer risk in pediatric patients and reported that thyroid exposure during a CT scan is related to an increased risk of cancer induction [18]. Tipnis et al. report that the thyroid dose acts as a secondary risk factor for thyroid cancer, with age and sex playing essential roles [19]. These findings agreed with those in our study. Although we focused on the short-term effects of radiation on non-cancer thyroid diseases, we also found an association between age, sex, and non-cancer thyroid diseases. Our study showed that a head CT scan was not associated with an increased incidence or risk of non-cancer thyroid disease.

There are different types of noncancer thyroid diseases, all of which have been adequately studied as risk factors of thyroid cancer. In their observational study, Nurdan et al. demonstrated a higher prevalence of differentiated thyroid cancer in patients with subacute thyroiditis [20]. Chen et al. revealed the risk of thyroid cancer in a population with Grave’s disease in a nationwide cohort study [21]. Chen et al. further revealed that patients with Hashimoto’s thyroiditis are at a higher risk of thyroid cancer [22]. Hence, we performed a subgroup analysis of three subtypes based on ICD-9 codes: simple and unspecified, nontoxic nodular goiter; thyrotoxicosis with or without goiter; and acquired hypothyroidism and thyroiditis. So, the purpose of this subgroup analysis was to determine how the radiation exposure of CT scan with a lower dose of radiation, would affect the thyroid function. As shown in Table 4, none of the subgroups displayed any increased risk with CT scan exposure with a non-significant change in the hypothyroidism group.

Radiation shielding is a strategy for reducing the radiation dose to an organ. Hopper et al. first introduced radiation shielding for the eyes, thyroid, and breasts for patients undergoing CT scans [23]. Abuzaid et al. demonstrated that thyroid shielding significantly decreases the dose to the thyroid during a head CT scan [24]. These findings do not conflict with ours because we only focused on non-cancer thyroid diseases and did not evaluate cancer risk. However, current studies consistently provided evidence of increased cancer risk with thyroiditis. Thyroid shielding may still contribute to reducing the exposure of radiation, thus reducing the risk of thyroid cancer. Although our study showed no increased risk in the CT scan group, female patients showed significant increased risk, which may have benefited from the strategy of radiation shielding. Further research should be designed and performed in this study setting to confirm its use in clinical practice.

This study had some limitations. First, the NHIRD did not contain information on clinical parameters, such as the Glasgow Coma Scale score or blood pressure. These factors may have influenced the decision to perform a CT scan. Second, we did not have access to potentially relevant personal behavioral information, such as smoking, alcohol consumption, or body mass index, which may have affected the results of thyroid diseases. Third, we only had a maximum of five years for observation; the latency period of the thyroid effect might be longer than our follow-up duration. Finally, the radiation dose may differ with different CT scan manufacturers and setting protocols, which may have influenced our study results. Therefore, further research is required to explore the risk of thyroid cancer with head CT scans.

## 5. Conclusions

In summary, this population-based cohort study revealed that CT scans in patients with minor head injuries was not associated with an increased incidence or risk of noncancer thyroid disease. The short-term adverse effects on the thyroid could be mild when a regular CT scan was appropriately conducted in Taiwan’s healthcare setting as per the radiation protection policy. Our findings may alleviate clinical concerns about thyroid diseases when arranging a head CT scan for patients with minor head injuries.

## Figures and Tables

**Table 1:** Baseline characteristics of CT scan and non-CT *-can patients with minor head injuries.

Variable	Comparison Group n = 34,684	CT Scan Group n = 14,041	*p* Value
Age			<0.0001
0–18	9479 (27.33%)	2075 (14.78%)	
19–45	14,214 (40.98%)	4831 (34.41%)	
46–65	6983 (20.13%)	3477 (24.76%)	
>65	4008 (11.56%)	3658 (26.05%)	
Sex			0.0291
Female	15,529 (44.77%)	6439 (45.86%)	
Male	19,155 (55.23%)	7602 (54.14%)	
Co-morbidities			
Diabetes mellitus	2320 (6.69%)	1864 (13.28%)	<0.0001
Hypertension	4775 (13.77%)	3729 (26.56%)	<0.0001
COPD **	3335 (9.62%)	1797 (12.80%)	<0.0001
Coronary artery disease	1462 (4.22%)	1282 (9.13%)	<0.0001
Cancer	669 (1.93%)	498 (3.55%)	<0.0001
Hospital stays (days) within 1 month			<0.0001
0	31,292 (90.22%)	9638 (68.64%)	
1–7	2727 (7.86%)	3364 (23.96%)	
8–14	665 (1.92%)	1039 (7.40%)	
Frequency of CT scan within 14 days			-
1	0 (0%)	13,217 (94.13%)	
≥2	0 (0%)	824 (5.87%)	
Frequency of CT scan within 2 months			-
1	5 (0.01%)	13,112 (93.38%)	
≥2	0 (0%)	929 (6.62%)	
Mortality within 6 months	307 (0.89%)	219 (1.56%)	<0.0001

* Computed tomography scan and non-computed tomography scan. ** Chronic obstructive pulmonary disease.

**Table 2:** Cumulative incidence rate of thyroid diseases.

	Follow-Up Person-Months	Case of Thyroid Diseases	Incidence Rate *(95% CI)	IRR
Comparison group	1,076,856	539	0.50 (0.46–0.54)	1
CT scan group	386,042	213	0.55 (0.48–0.63)	1.10 (0.94–1.29)
1 time	362,352	198	0.55 (0.48–0.63)	1.10 (0.94–1.29)
≥2 times	23,690	15	0.63 (0.38–1.05)	1.09 (0.93–1.28)

* per 1000 person-months.

**Table 3:** Results of Cox proportional hazards analysis.

Variable	aHR	95% CI	*p*-Value
CT exposure			
Comparisons	Reference	-	-
1 time	0.933	0.787–1.107	0.4286
≥2 times	1.053	0.618–1.794	0.8496
Age			
0–18	Reference	-	-
19–45	0.666	0.534–0.829	0.0003
46–65	1.096	0.902–1.331	0.3567
<65	1.065	0.830–1.367	0.6187
Sex			
Female	Reference	-	-
Male	0.383	0.327–0.447	<0.0001
Co-morbidities			
Diabetes mellitus	1.246	0.975–1.593	0.0789
Hypertension	1.102	0.878–1.384	0.4027
COPD	1.511	1.216–1.877	0.0002
Coronary artery disease	1.168	0.869–1.569	0.3029
Cancer	1.716	1.187–2.480	0.0041
Hospital stays (days) within 1 month			
0	Reference	-	-
1–7	1.000	0.802–1.245	0.9973
8–14	1.365	0.977–1.907	0.0679

**Table 4:** Risk of subtypes of thyroid diseases in the CT scan group.

	aHR (95% C.I.)		
		CT Exposure	
Diagnosis	Comparisons	1 time	≥2 times
Thyroid disease (ICD-9 240–246)	Reference	0.933 (0.787–1.107)	1.053 (0.618–1.794)
Simple and unspecified, nontoxic nodular goiter (ICD-9 240–241)	Reference	0.928 (0.698–1.235)	0.769 (0.241–2.457)
Thyrotoxicosis with or without goiter (ICD-9 242)	Reference	0.850 (0.603–1.200)	0.755 (0.232–2.457)
Acquired hypothyroidism, thyroiditis (ICD-9 244, 245)	Reference	1.104 (0.758–1.608)	1.796 (0.699–4.619)
